# The relationship between sedentary behaviour and physical literacy in Canadian children: a cross-sectional analysis from the RBC-CAPL Learn to Play study

**DOI:** 10.1186/s12889-018-5892-9

**Published:** 2018-10-02

**Authors:** Travis J. Saunders, Dany J. MacDonald, Jennifer L. Copeland, Patricia E. Longmuir, Joel D. Barnes, Kevin Belanger, Brenda Bruner, Melanie J. Gregg, Nathan Hall, Angela M. Kolen, Barbi Law, Luc J. Martin, Dwayne Sheehan, Michelle R. Stone, Sarah J. Woodruff, Mark S. Tremblay

**Affiliations:** 1 Department of Applied Human Sciences, University of Prince Edward Island, 550 University Avenue, Charlottetown, PE C1A 4P3 Canada; 2 Department of Kinesiology and Physical Education, University of Lethbridge, Lethbridge, AB T1K 3M4 Canada; 3 Healthy Active Living and Obesity (HALO) Research Group, Children’s Hospital of Eastern Ontario Research Institute, Ottawa, ON K1H 8L1 Canada; 4 School of Physical and Health Education, Nipissing University, North Bay, ON P1B 8L7 Canada; 5 Department of Kinesiology and Applied Health, University of Winnipeg, Winnipeg, MB R3B 2E9 Canada; 6 Department of Human Kinetics, St. Francis Xavier University, Antigonish, NS B2G 0W5 Canada; 7 School of Kinesiology and Health Studies, Queen’s University, Kingston, ON K7L 3N6 Canada; 8 Faculty of Health, Community and Education, Mount Royal University, Calgary, AB T3E 6K6 Canada; 9 School of Health and Human Performance, Dalhousie University, Halifax, NS B3H 4R2 Canada; 10 Department of Kinesiology, University of Windsor, Windsor, ON N9B 3P4 Canada

**Keywords:** Physical literacy, Sedentary behaviour, Screen time, TV, Computer, Video game, Reading, Physical activity

## Abstract

**Background:**

Physical literacy is the foundation of a physically active lifestyle. Sedentary behaviour displays deleterious associations with important health indicators in children. However, the association between sedentary behaviour and physical literacy is unknown. The purpose of this study was to identify the aspects of physical literacy that are associated with key modes of sedentary behaviour among Canadian children participating in the RBC-CAPL Learn to Play study.

**Methods:**

A total of 8,307 children aged 8.0-12.9 years were included in the present analysis. Physical literacy was assessed using the Canadian Assessment of Physical Literacy, which measures four domains (Physical Competence, Daily Behaviour, Motivation and Confidence, Knowledge and Understanding). Screen-based sedentary behaviours (TV viewing, computer and video game use), non-screen sedentary behaviours (reading, doing homework, sitting and talking to friends, drawing, etc.) and total sedentary behaviour were assessed via self-report questionnaire. Linear regression models were used to determine significant (*p<*0.05) correlates of each mode of sedentary behaviour.

**Results:**

In comparison to girls, boys reported more screen time (2.7±2.0 vs 2.2±1.8 hours/day, Cohen’s *d*=0.29), and total sedentary behaviour (4.3±2.6 vs 3.9±2.4 hours/day, Cohen’s *d*=0.19), but lower non-screen-based sedentary behaviour (1.6±1.3 vs 1.7±1.3 hours/day, Cohen’s *d=*0.08) (all *p*< 0.05). Physical Competence (standardized β’s: -0.100 to -0.036, all *p<*0.05) and Motivation and Confidence (standardized β’s: -0.274 to -0.083, all *p<*0.05) were negatively associated with all modes of sedentary behaviour in fully adjusted models. Knowledge and Understanding was negatively associated with screen-based modes of sedentary behaviour (standardized β’s: -0.039 to -0.032, all *p<*0.05), and positively associated with non-screen sedentary behaviour (standardized β: 0.098, *p<*0.05). Progressive Aerobic Cardiovascular Endurance Run score and log-transformed plank score were negatively associated with all screen-based modes of sedentary behaviour, while the Canadian Agility and Movement Skill Assessment score was negatively associated with all modes of sedentary behaviour other than TV viewing (all *p<*0.05).

**Conclusions:**

These results highlight differences in the ways that screen and non-screen sedentary behaviours relate to physical literacy. Public health interventions should continue to target screen-based sedentary behaviours, given their potentially harmful associations with important aspects of physical literacy.

## Background

Sedentary behaviour (SB) refers to any behaviour done with a low energy expenditure (≤1.5 METs [metabolic equivalents]) while sitting, reclining, or lying down [1]. SBs are increasingly prevalent among children in developed nations [2, 3], and are deleteriously associated with numerous physical and psycho-social health indicators in pediatric populations [4–6]. A recent systematic review by Carson et al. [4] concluded that children who accumulate large amounts of SB, and especially screen-based SBs (e.g., television, computer and video games), tended to have unfavourable measures of body composition, cardiometabolic risk, physical fitness, behavioural conduct/prosocial behaviour and self-esteem. It has also been noted that the associations between SBs and health differ based on the modality of SB; screen-based SBs tend to be adversely associated with health indicators, while non-screen-based SBs (e.g., reading) typically display a neutral or even beneficial association with health [2, 4].

Physical literacy (PL) is defined as “the motivation, confidence, physical competence, knowledge and understanding to value and take responsibility for engagement in physical activities for life” [7]. PL has gained increasing attention in recent years, and has been suggested as the foundation for lifelong healthy active living [8, 9]. Although recent studies have investigated behavioural and socio-demographic correlates of SB [10–15], to date no study has examined the relationship between SB and key components of PL. If there is a relationship between SB and PL, this could provide support for the role of PL in promoting a healthy active lifestyle.

The Canadian Assessment of Physical Literacy (CAPL) is a valid tool for measuring PL in children aged 8-12 years, and provides information on four key domains: Daily Behaviour, Physical Competence, Knowledge and Understanding, and Motivation and Confidence [9]. Within the Daily Behaviour domain, the CAPL also measures self-reported TV, computer, and non-screen SB, as well as total SB. In 2014-2016, more than 10,000 children from 11 sites across Canada were included as part of the Royal Bank of Canada–Canadian Assessment of Physical Literacy (RBC-CAPL) Learn to Play study [9]. The purpose of this analysis was to determine if aspects of PL were associated with key modes of SB among participating children.

We hypothesized that all forms of SB would be negatively associated with overall PL, and with individual PL domains. With respect to individual CAPL components, we hypothesized that SB would be positively associated with age and markers of adiposity, and negatively associated with measures of aerobic and musculoskeletal fitness, self-reported physical activity, and the maximum amount of time that participants felt that children in general should spend in front of a screen on a daily basis. Finally, we hypothesized that PL would be more strongly associated with screen-based modes of SB, in comparison to non-screen SB.

## Methods

### Participants and study design

Full details of the CAPL, and the RBC-CAPL Learn to Play study, are available via the CAPL website [16] and in previous publications [9, 17]. Briefly, the CAPL was developed by the Healthy Active Living and Obesity Research Group at the Children’s Hospital of Eastern Ontario Research Institute. The CAPL includes eight tests of Physical Competence (outlined below), a 21-item Knowledge and Understanding self-report questionnaire, and the 17-item Children’s Self-Perception of Adequacy in and Predilection for Physical Activity (CSAPPA) [18]. Participants were also asked to wear a pedometer for 7 days to measure daily step counts. All tests were administered by trained staff. The validity and reliability of individual CAPL components have been published previously [18–23].

The present analysis was coordinated by the Healthy Active Living and Obesity Research Group at the Children’s Hospital of Eastern Ontario Research Institute, and data were collected from 11 sites in 7 Canadian provinces (Table 1). Training of staff at the individual sites took place in May of 2014, and data collection ended in January of 2017. To be included, participants had to be between 8.0 and 12.9 years of age at the time of data collection. Participants were excluded from the study if they had been told by their physician to avoid exercise. There were no other exclusion criteria. Participants were recruited through public and private schools, camps, community recreation centres and after-school programs. Study procedures were approved by research ethics boards at the Children’s Hospital of Eastern Ontario Research Institute as well as each individual CAPL site, and by participating school boards and community organizations. Written informed consent was obtained from parents or legal guardians, while assent was obtained from all study participants.

**Table 1:** Sedentary behaviours across RBC-CAPL study sites (*n*=8307)

CAPL site	n (boy/girl)	BMI *z*-score	TV time (hours/day)	Computer/video game time (hours/day)	Total screen time (hours/day)	Meeting screen time guidelines (%)	Non-screen sedentary time (hours/day)	Total sedentary time (hours/day)
Antigonish, Nova Scotia	832 (403/429)	0.9 (1.3)	1.3 (1.2)	1.2 (1.2)	2.4 (2.1)	56.4	1.5 (1.3)	4.0 (2.8)
Halifax, Nova Scotia	639 (313/326)	0.4 (1.2)	0.9 (0.9)	0.9 (1.0)	1.8 (1.7)	70.4	1.5 (1.2)	3.4 (2.3)
Charlottetown, Prince Edward Island	442 (218/224)	0.6 (1.2)	1.1 (1.0)	1.0 (1.1)	2.0 (1.7)	61.5	1.7 (1.3)	3.7 (2.3)
Trois-Rivières, Québec	42 (27/15)	-0.01 (1.2)	1.1 (0.9)	1.1 (1.2)	2.2 (1.5)	59.5	1.7 (1.3)	3.9 (1.9)
Ottawa, Ontario	516 (258/258)	0.7 (1.3)	1.4 (1.1)	1.2 (1.2)	2.6 (1.9)	49.8	1.6 (1.3)	4.2 (2.5)
North Bay, Ontario	1041 (489/552)	0.7 (1.2)	1.3 (1.1)	1.3 (1.3)	2.6 (2.1)	52.5	1.7 (1.3)	4.3 (2.7)
Windsor, Ontario	1166 (612/554)	0.6 (1.3)	1.4 (1.2)	1.4 (1.3)	2.8 (2.0)	47.8	1.6 (1.3)	4.4 (2.5)
Winnipeg, Manitoba	1072 (525/547)	0.6 (1.3)	1.3 (1.2)	1.4 (1.2)	2.7 (2.0)	47.7	1.6 (1.2)	4.3 (2.6)
Lethbridge, Alberta	907 (454/453)	0.4 (1.2)	1.2 (1.1)	1.1 (1.2)	2.3 (1.8)	56.1	1.7 (1.3)	4.0 (2.3)
Calgary, Alberta	1207 (605/602)	0.4 (1.3)	1.2 (1.0)	1.2 (1.1)	2.4 (1.8)	53.9	1.8 (1.3)	4.2 (2.3)
Victoria, British Columbia	443 (239/204)	0.5 (1.2)	1.2 (1.0)	1.0 (1.0)	2.2 (1.7)	60.0	1.8 (1.3)	4.0 (2.2)
Overall	8307 (4143/4164)	0.7 (1.3)	1.2 (1.1)	1.2 (1.2)	2.4 (1.9)	54.3	1.3 (1.3)	4.1 (2.5)

Data are presented as mean (SD)

Meeting screen time guidelines was defined as accumulating ≤2 hours/day of recreational screen time

*BMI* body mass index, *CAPL* Canadian Assessment of Physical Literacy

### Sedentary behaviour

Participants were asked to self-report the time spent watching TV, playing video or computer games or using a computer for non-school work, and time spent sitting down doing non-screen-based activities outside of school time (e.g., reading a book, doing homework, sitting and talking to friends, drawing, etc.). Response categories for each question were “I did not spend time” = 0 hours/day, “Less than 1 hour” = 0.5 hours/day, “1 hour” = 1 hour/day, “2 hours” = 2 hours/day, “3 hours” = 3 hours/day, “4 hours” = 4 hours/day, and “5 or more hours” = 5 hours/day. Each question was asked for a typical school day and also for a typical weekend day. A weighted mean of daily time (decimal hours) spent in each mode of SB was calculated using the following equation: [(hours of SB on school days x 5) + (hours of SB on weekends x 2)]/7 [11, 12]. Average daily TV and computer/video game time were summed to calculate total screen time. TV, computer/video game, and non-screen SBs were summed to calculate total SB. Participants were also asked, “What is the most time that children should look at a screen each day?”, with response options of 30 minutes, 1 hour, 2 hours, and 4 hours.

### Potential correlates of SB

#### Demographic characteristics

Age and gender were self-reported by participants. CAPL site and season of data collection were recorded by researchers. Seasons were identified as follows: winter = 1, spring = 2, summer = 3, and fall = 4.

#### Daily Behaviour

Using a self-report questionnaire, children were asked, “During the past week (7 days), on how many days were you physically active for a total of at least 60 minutes per day?” and asked to select an option between 0 and 7. Physical activity (PA) was also assessed via SC-StepRx pedometer (StepsCount, Deep River, ON, Canada) [19, 23]. Participants were asked to record the number of steps they took, the time the pedometer was put on in the morning and taken off at night, and the amount of missing hours, in a tracking log for seven consecutive days. Pedometer data were considered valid if the number of steps fell between 1,000 and 30,000 steps/day, with at least 10 hours of wear-time. For pedometer data to be included, participants were required to have ≥3 valid days, with no minimum requirement for week or weekend days [24, 25].

Typically the Daily Behaviour score is calculated out of 32 points, based on the pedometer data, self-reported PA, and SB-related questions [9]. However, for the purposes of this analysis, Daily Behaviour was re-calculated after removing SB-related questions, for a maximum score of 24 points. It was also possible to calculate a Daily Behaviour score using only self-reported PA data if a participant did not have valid pedometer data, which was the case for roughly half of study participants. Because of the large proportion of missing pedometer data, daily steps were not included as an individual variable in other analyses to maximize sample size. Given that the re-calculated Daily Behaviour score was based solely on self-reported PA for many participants in the present analysis, this domain was not included on its own in regression analyses, although it was used to calculate total PL.

#### Physical Competence

Physical Competence was assessed by trained study staff using established methods. Motor skills were measured using the Canadian Agility and Movement Skill Assessment (CAMSA) as described by Longmuir et al. [21]. Muscular endurance was assessed using the prone plank, following the protocol validated in this population by Boyer et al. [20]. Handgrip strength, sit-and-reach flexibility, and waist circumference were assessed according to Canadian Society for Exercise Physiology protocols [26]. Aerobic fitness was assessed using the Progressive Aerobic Cardiovascular Endurance Run (PACER) shuttle run [22]. Body mass index (BMI) *z*-scores were calculated using directly measured height and weight and World Health Organization growth curves [27]. Participants also received an overall Physical Competence score based on the above indicators, with a maximum of 32 total points [9].

#### Knowledge and Understanding

Knowledge and Understanding were assessed via self-report questionnaire, and a score was calculated out of a possible 18 points [9]. The content of this questionnaire was meant to capture Canadian provincial curricula for physical and health education in grades 4, 5, and 6: awareness of fitness terminology; perceptions of health; use of safety equipment during PA; and basic methods on how to improve fitness levels [9].

#### Motivation and Confidence

The Motivation and Confidence domain was assessed via self-report questionnaire. Children were asked to rate their agreement on a scale of 1 (disagree a lot) to 5 (agree a lot) to questions describing PA barriers and benefits (10 and 9 items, respectively) [28]. A benefits-to-barriers ratio was calculated as the perceived benefits minus perceived barriers of PA. Adequacy and predilection subscale scores were used from 16 items taken from the CSAPPA Scale [18]. Finally, PA and skill level compared to others were self-reported with one item each, using a scale from 1 (a lot less active; others are better) to 10 (a lot more active; I’m a lot better).

#### Overall physical literacy score

Based on their performance in each of the four individual CAPL domains, participants received an overall PL score out of a maximum of 100 points [9]. For all analyses in the present manuscript, the total PL score was re-calculated after removal of all SB items.

### Statistical analyses

Statistical analyses were conducted using SPSS 25 (IBM SPSS Statistics, New York, NY, USA). All variables were normally distributed except for plank score, which was log transformed. Unpaired t-tests and chi-square tests were used to assess differences between boys and girls, and between those with complete data compared to those with missing data. Correlations between each mode of SB were assessed via Pearson *r*. Effect sizes were determined using Cohen’s *d*.

Multi-level models were examined with participants nested within sites; however, considering that the variance explained by the sites was <2.5% for all modes of SB, linear regression models were used to determine correlates of each mode of SB instead. Potential correlates were initially identified using minimally adjusted models, adjusted only for age and gender. Separate models were run for each potential correlate. Any correlates associated with a mode of SB in the minimally adjusted models (*p<*0.10) were included in the fully adjusted model for that SB. Fully adjusted models were run twice for each mode of SB: once for CAPL domain scores (Knowledge and Understanding, Physical Competence, and Motivation and Competence), and once for individual CAPL components (BMI *z*-score, PACER score, etc.). Variables that remained significant (*p*<0.05) in the fully adjusted linear regression model were considered correlates of that mode of SB. Analyses are presented for the total sample, and for boys and girls separately.

BMI *z*-score and waist circumference were highly correlated (*r*=0.800; *p<*0.001). Therefore, whenever both were significant in initial models for a given mode of SB, the variable accounting for the greater proportion of variance was included in the fully adjusted model to avoid collinearity. In all cases where both were significant, waist circumference was included in the fully adjusted models.

## Results

The full sample included 10,034 participants (5,004 boys, 5,030 girls); 1,727 were missing data for at least one variable of interest, and were excluded from subsequent analyses (Table 2). Although there were several statistically significant differences between those with missing and those with complete data, the effect sizes were small (i.e., ≤0.15 for all differences). In comparison to participants with complete data, those with missing data were younger (10.5±1.2 vs 10.6±1.2 years, Cohen’s *d*=0.08, *p*=0.040); heavier (BMI *z*-score 0.7±1.3 vs 0.6±1.3, Cohen’s *d*=0.08, *p=*0.027); reported lower frequency of PA (4.9±2.1 vs 5.0±1.9 days/week, Cohen’s *d*=0.06, *p=*0.034); and had lower scores for sit and reach (27.6±8.6 vs 28.2±8.4 cm, Cohen’s *d*=0.07, *p=*0.025), PACER (22.2±13.2 vs 23.5±14.2 laps completed, Cohen’s *d*=0.09, *p=*0.002), CAMSA (20.2±4.2 vs 20.7±3.8, Cohen’s *d*=0.12, *p<*0.001), and the Motivation and Confidence (12.2±3.0 vs 12.5±2.7, Cohen’s *d*=0.11, *p=*0.001), Physical Competence (18.9±5.1 vs 19.7±4.3, Cohen’s *d*=0.15, *p<*0.001), and Knowledge and Understanding (11.7±2.7 vs 12.1±2.7, Cohen’s *d=*0.15, *p<*0.001) domains of PL.

**Table 2:** Participants with missing data

Variable	Missing data (*n*)
Age	105
Sit and reach	414
Handgrip	366
PACER	641
Plank	428
Waist circumference	639
BMI *z*-score	622
Frequency of PA	247
TV time	259
Computer time	262
Screen time	264
Non-screen SB	258
Total SB	522
Physical Competence	646
Motivation and Confidence	409
Knowledge and Understanding	237
CAPL score	703
CAMSA	546

Some participants were missing data for multiple variables; 1727 participants were missing data for at least one variable

*BMI* body mass index, *CAMSA* Canadian Agility and Movement Skill Assessment *CAPL* Canadian Assessment of Physical Literacy, *PA* physical activity, *PACER* Progressive Aerobic Cardiovascular Endurance Run, *SB* sedentary behaviour.

Descriptive characteristics of study participants are presented in Table 3. Site sample sizes ranged from 42 participants (Trois-Rivières, Québec) to 1,207 participants (Calgary, Alberta). Participants had an average age of 10.6 years, and reported accumulating 2.4 hours/day of screen time, and 4.1 hours/day of total SB. Overall, 54.3% of participants reported meeting Canadian guidelines for recreational screen time (≤2 hours/day), ranging from a low of 47.7% (Winnipeg, Manitoba) to a high of 70.4% (Halifax, Nova Scotia).

**Table 3:** Participant characteristics (*n*=8307)

Variables	Mean (SD)
CAPL domain scores	
Total Physical Literacy score (/100)	62 (11.0)
Knowledge and Understanding score (/18)	12.1 (2.7)
Motivation and Confidence score (/18)	12.5 (2.7)
Physical Competence score (/32)	19.7 (4.3)
Individual CAPL components	
Age (years)	10.6 (1.2)
BMI *z*-score	0.6 (1.3)
Waist circumference (cm)	67.2 (10.7)
Most time children should spend in front of a screen (hours/day)	0.9 (0.4)
Frequency of PA (days/week)	5.0 (1.9)
Plank (seconds)	61.8 (43.3)
Sit and reach (cm)	28.2 (8.4)
Grip strength (kg)	33.6 (9.4)
CAMSA score	20.7 (3.8)
PACER (laps completed)	23.5 (14.2)
Participants tested in each season (n)	
Winter	1889
Spring	3122
Summer	1330
Fall	1966

Data presented as mean (SD)

*BMI* body mass index, *CAMSA* Canadian Agility and Movement Skill Assessment, *CAPL* Canadian Assessment of Physical Literacy, *PA* physical activity, *PACER* Progressive Aerobic Cardiovascular Endurance Run

**Fig. 1:**
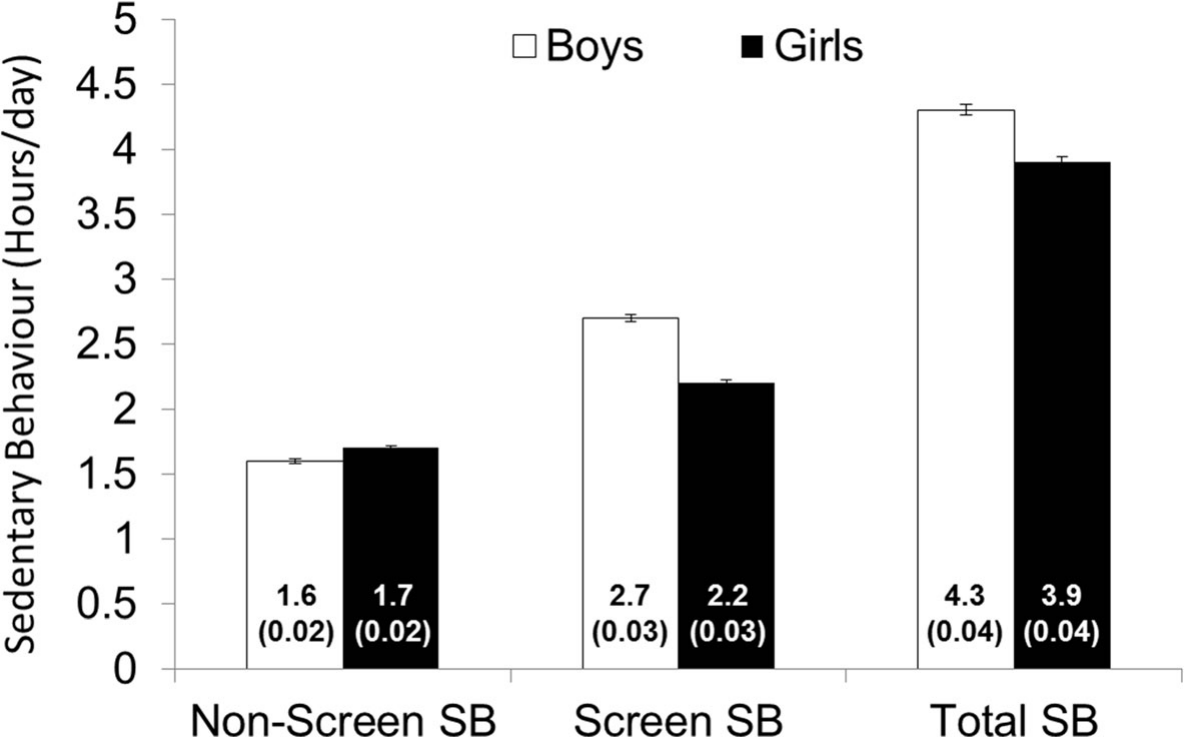
Daily sedentary behaviour in boys and girls. Data are presented as mean (standard error). Values were significantly different between boys and girls for all modes of sedentary behaviour. SB: sedentary behaviour

Differences in sedentary behaviour were small between boys and girls, although boys had higher scores for TV (1.3±1.2 vs 1.2±1.0 hours/day, Cohen’s *d*=0.10), computer/video game use (1.4±1.3 vs 1.0±1.1 hours/day, Cohen’s *d*=0.39), total screen time (2.7±2.0 vs 2.2±1.8 hours/day, Cohen’s *d*=0.29), and total SB (4.3±2.6 vs 3.9±2.4 hours/day, Cohen’s *d*=0.19), and lower scores for non-screen-based SB (1.6±1.3 vs 1.7±1.3 hours/day, Cohen’s *d=*0.08) (all *p<*0.05) (Figure 1). Girls were more likely than boys to meet Canada’s screen time guidelines (61% vs 48%, respectively, *p<*0.05). There was a moderate positive association between TV and computer/video game use (*r*=0.40), with trivial associations between non-screen SB and TV (*r=*0.15), computer/video game use (*r*=0.17), or total screen time (*r*=0.19) (all *p<*0.05; Table 4).

**Table 4:** Pearson correlation coefficients for different modes of sedentary behaviour (*n*=8307)

	TV time	Computer/video game time	Total screen time	Total non-screen time	Total sedentary behaviour
TV time	1				
Computer/video game time	0.40	1			
Total screen time	0.82	0.85	1		
Total non-screen time	0.15	0.17	0.19	1	
Total sedentary behaviour	0.71	0.74	0.86	0.66	1

All *p<*0.05

### Correlates in minimally adjusted models

Results from the minimally adjusted models are presented in Tables 5, 6, 7, 8 and 9. Although all PL domains were significantly associated with each mode of SB in the full sample, Motivation and Confidence (standardized β’s: -0.300 to -0.078) and total PL (standardized β’s: -0.272 to -0.038) exhibited stronger associations with all modes of SB than Knowledge and Understanding (standardized β’s: -0.110 to 0.074) or Physical Competence (standardized β’s: -0.204 to -0.050) (all *p<*0.05). Among individual CAPL components, the self-reported maximum amount of time that participants felt children should spend in front of a screen each day was positively associated with all modes of SB (standardized β’s: 0.114 to 0.421, all *p<*0.05). Gender, frequency of self-reported PA, PACER score, log-transformed plank score, and CAMSA score were all consistently associated with screen-based modes of SB. Season of data collection was the only investigated correlate that did not have a significant association with TV, computer, total screen, and total SB when investigating both genders combined, or when examining boys separately (all *p*>0.10).

**Table 5:** Correlates of TV viewing (*n*=8307)

Variable	All Participants			Boys (n=4143)			Girls (n=4164)		
	Unstandardized β (95% CI)	Standardized β	*p* value	Unstandardized β (95% CI)	Standardized β	*p* value	Unstandardized β (95% CI)	Standardized β	*p* value
Minimally adjusted models									
CAPL domain scores									
Total Physical Literacy	**-0.019 (-0.021; -0.017)**	**-0.188**	**<0.001**	**-0.022 (-0.025; -0.018)**	**-0.213**	**<0.001**	**-0.015 (-0.18; -0.12)**	**-0.153**	**<0.001**
Knowledge and Understanding score	**-0.033 (-0.042; -0.024)**	**-0.081**	**<0.001**	**-0.039 (-0.052; -0.026)**	**-0.095**	**<0.001**	**-0.025 (-0.038; -0.013)**	**-0.063**	**<0.001**
Motivation and Confidence score	**-0.077 (-0.085; -0.068)**	**-0.187**	**<0.001**	**-0.085 (-0.097; -0.072)**	**-0.203**	**<0.001**	**-0.067 (-0.079; -0.055)**	**-0.165**	**<0.001**
Physical Competence score	**-0.039 (-0.044; -0.033)**	**-0.150**	**<0.001**	**-0.045 (-0.052; -0.037)**	**-0.176**	**<0.001**	**-0.031 (-0.039; -0.023)**	**-0.117**	**<0.001**
Individual CAPL components									
Age (years)	**0.041 (0.021; 0.061)**	**0.044**	**<0.001**	0.017 (-0.013; 0.047)	0.017	0.264	**0.065 (0.038; 0.092)**	**0.074**	**<0.001**
Gender (boy=1, girl=2)	**-0.107 (-0.154; -0.060)**	**-0.048**	**<0.001**	-	-	-	-	-	-
CAPL site	**-0.00002 (-0.00004; -0.00001)**	**-0.029**	**0.009**	**-0.00003 (-0.0006; -0.000007)**	**-0.038**	**0.015**	-0.0002 (-0.0004; -0.00001)	-0.019	0.230
Season	0.012 (-0.010; 0.034)	0.012	0.291	0.002 (-0.031; 0.035)	0.002	0.888	0.020 (-0.009; 0.050)	0.021	0.175
BMI *z*-score	**0.047 (0.028; 0.065)**	**0.054**	**<0.001**	**0.056 (0.29; 0.82)**	**0.064**	**<.0001**	**0.037 (0.011; 0.063)**	**0.043**	**0.005**
Waist circumference (cm)	**0.007 (0.005; 0.010)**	**0.071**	**<0.001**	**0.008 (0.004; 0.011)**	**0.072**	**<0.001**	**0.007 (0.004; 0.010)**	**0.072**	**<0.001**
Most time children should spend in front of a screen (hours/day)	**0.007 (0.006; 0.007)**	**0.303**	**<0.001**	**0.007 (0.006; 0.007)**	**0.308**	**<0.001**	**0.007 (0.007; 0.008)**	**0.292**	**<0.001**
Frequency of PA (days/week)	**-0.058 (-0.071; -0.046)**	**-0.101**	**<0.001**	**-0.056 (-0.074; -0.038)**	**-0.095**	**<0.001**	**-0.061 (-0.078; -0.044)**	**-0.107**	**<0.001**
Ln plank	**-0.255 (-0.297; -0.212)**	**-0.128**	**<0.001**	**-0.281 (-0.343; -0.219)**	**-0.137**	**<0.001**	**-0.225 (-0.283; -0.167)**	**-0.117**	**<0.001**
Sit and reach (cm)	**-0.007 (-0.010; -0.004)**	**-0.053**	**<0.001**	**-0.011 (-0.016; -0.007)**	**-0.074**	**<0.001**	**-0.004 (-0.008; 0.00003)**	**-0.030**	**0.052**
Grip strength (kg)	**-0.005 (-0.008; -0.002)**	**-0.042**	**0.001**	**-0.007 (-0.011; -0.003)**	**-0.059**	**0.001**	-0.002 (-0.006; 0.001)	-0.021	0.224
CAMSA score	**-0.031 (-0.038; -0.025)**	**-0.109**	**<0.001**	**-0.041 (-0.051; -0.032)**	**-0.0138**	**<0.001**	**-0.021 (-0.030; -0.012)**	**-0.075**	**<0.001**
PACER (laps completed)	**-0.011 (-0.013; -0.009)**	**-0.139**	**<0.001**	**-0.012 (-0.014; -0.009)**	**-0.161**	**<0.001**	**-0.009 (-0.012; -0.006)**	**-0.101**	**<0.001**
Fully adjusted models									
CAPL domain scores									
Knowledge and Understanding score	**-0.014 (-0.023; -0.005)**	**-0.034**	**0.003**	**-0.018 (-0.031; -0.005)**	**-0.044**	**0.007**	-0.009 (-0.022; 0.004)	-0.023	0.161
Motivation and Confidence score	**-0.061 (-0.070; -0.051)**	**-0.148**	**<0.001**	**-0.064 (-0.077; -0.050)**	**-0.153**	**<0.001**	**-0.057 (-0.070; -0.044)**	**-0.140**	**<0.001**
Physical Competence score	**-0.023 (-0.029; -0.017)**	**-0.088**	**<0.001**	**-0.027 (-0.036; -0.019)**	**-0.108**	**<0.001**	**-0.016 (-0.025; -0.008)**	**-0.062**	**<0.001**
Individual CAPL components									
Age (years)	0.023 (-0.0001; 0.046)	0.024	0.051	0.0002 (-0.034; 0.034	0.0002	0.989	**0.043 (0.012; 0.073)**	**0.048**	**0.006**
Gender (boy=1, girl=2)	-0.40 (-0.089; 0.010)	-0.018	0.118	-	-	-	-	-	-
CAPL site	-0.00001 (-0.00003; 0.000004)	-0.016	0.126	-0.00002 (-0.00004; 0.00001)	-0.019	0.206	-0.00001 (-0.00003; 0.00001)	-0.013	0.397
Waist circumference (cm)	0.003 (-0.0002; 0.005)	0.024	0.065	0.001 (-0.003; 0.005)	0.006	0.763	**0.005 (0.001; 0.009)**	**0.049**	**0.009**
Most time children should spend in front of a screen (hours/day)	**0.006 (0.006; 0.007)**	**0.284**	**<0.001**	**0.006 (0.005; 0.007)**	**0.283**	**<0.001**	**0.007 (0.006; 0.008)**	**0.282**	**<0.001**
Frequency of PA (days/week)	**-0.031 (-0.043; -0.019)**	**-0.053**	**<0.001**	**-0.020 (-0.038; -0.002)**	**-0.034**	**0.028**	**-0.043 (-0.059; -0.026)**	**-0.076**	**<0.001**
Ln plank	**-0.107 (-0.155; -0.058)**	**-0.054**	**<0.001**	**-0.090 (-0.162; -0.019)**	**-0.044**	**0.014**	**-0.124 (-0.190; -0.058)**	**-0.065**	**<0.001**
Sit and reach	-0.0002 (-0.003; 0.003)	-0.001	0.914	-0.004 (-0.009; 0.001)	-0.026	0.093	0.003 (-0.001; 0.007)	0.021	0.173
Grip strength (kg)	-0.001 (-0.004; 0.002)	-0.009	0.502	0.001 (-0.004; 0.006)	0.008	0.665	-0.004 (-0.008; 0.0004)	-0.035	0.072
CAMSA score	-0.003 (-0.010; 0.004 )	-0.011	0.413	-0.007 (-0.018; 0.004)	-0.023	0.212	-0.00002 (-0.010; 0.010)	-0.0001	0.998
PACER (laps completed)	**-0.004 (-0.006; -0.002)**	**-0.051**	**<0.001**	**-0.005 (-0.008; -0.002)**	**-0.071**	**<0.001**	-0.002 (-0.005; 0.001)	-0.023	0.210

Minimally adjusted models adjusted for age and gender. Standardized β for age and gender in minimally adjusted models ranged from 0.024 to 0.081 and -0.074 to -0.035, respectively. Fully adjusted models included all variables significant (*p<*0.10) in minimally adjusted models (as well as age and gender in the model of CAPL domains). Separate fully adjusted models were run for CAPL domains and individual CAPL components. Bolded variables were significant <0.10 in minimally adjusted models, and <0.05 in fully adjusted models. The fully adjusted models of CAPL domain scores and CAPL components accounted for 5% and 11%, respectively, of the total variance in TV viewing in the full sample.

*BMI* body mass index, *CAMSA* Canadian Agility and Movement Skill Assessment, *CAPL* Canadian Assessment of Physical Literacy, *CI* confidence interval, *Ln*: natural log transformation, *PA* physical activity, *PACER* Progressive Aerobic Cardiovascular Endurance Run

**Table 6:** Correlates of computer and video game use (*n*=8307)

Variable	All participants			Boys (n=4143)			Girls (n=4164)		
	Unstandardized β (95% CI)	Standardized β	*p* value	Unstandardized β (95% CI)	Standardized β	*p* value	Unstandardized β (95% CI)	Standardized β	*p* value
Minimally adjusted models									
CAPL domain scores									
Total Physical Literacy	**-0.030 (-0.032; 0.027)**	**-0.272**	**<0.001**	**-0.036 (-0.039; -0.032)**	**-0.321**	**<0.001**	**-0.022 (-0.025; -0.019)**	**-0.215**	**<0.001**
Knowledge and Understanding score	**-0.045 (-0.055; -0.035)**	**-0.103**	**<0.001**	**-0.051 (-0.066; -0.037)**	**-0.114**	**<0.001**	**-0.038 (-0.051; -0.025)**	**-0.093**	**<0.001**
Motivation and Confidence score	**-0.138 (-0.147; -0.129)**	**-0.309**	**<0.001**	**-0.166 (-0.179; -0.153)**	**-0.362**	**<0.001**	**-0.105 (-0.118; -0.093)**	**-0.254**	**<0.001**
Physical Competence score	**-0.053 (-0.059; -0.047)**	**-0.190**	**<0.001**	**-0.063 (-0.071; -0.055)**	**-0.227**	**<0.001**	**-0.039 (-0.048; -0.031)**	**-0.145**	**<0.001**
Individual CAPL components									
Age (years)	**0.095 (0.073; 0.116)**	**0.093**	**<0.001**	**0.084 (0.051; 0.117)**	**0.078**	**<0.001**	**0.105 (0.078; 0.133)**	**0.116**	**<0.001**
Gender (boy=1, girl=2)	**-0.463 (-0.513; -0.412)**	**-0.193**	**<0.001**	-	-	-	-	-	-
CAPL site	**-0.00003 (-0.0004; -0.00005)**	**-0.026**	**0.014**	-0.00003 (-0.00006; -0.000001)	-0.030	0.055	-0.00002 (-0.0005; 0.000005)	-0.024	0.119
Season	-0.013 (-0.036; 0.011)	-0.011	0.290	-020 (-0.056; 0.017)	-0.017	0.290	-0.006 (-0.036; 0.024)	-0.006	0.677
BMI *z*-score	**0.044 (0.024; 0.064)**	**0.047**	**<0.001**	**0.044 (0.014; 0.073)**	**0.046**	**0.003**	**0.045 (0.018; 0.071)**	**0.050**	**0.001**
Waist circumference (cm)	**0.009 (0.006; 0.011)**	**0.078**	**<0.001**	**0.010 (0.006; 0.013)**	**0.083**	**<0.001**	**0.008 (0.005; 0.011)**	**0.076**	**<0.001**
Most time children should spend in front of a screen (hours/day)	**0.010 (0.009; 0.010)**	**0.398**	**<0.001**	**0.010 (0.009; 0.011)**	**0.426**	**<0.001**	**0.009 (0.009; 0.010)**	**0.364**	**<0.001**
Frequency of PA (days/week)	**-0.109 (-0.122; -0.096)**	**-0.173**	**<0.001**	**-0.120 (-0.139; -0.100)**	**-0.185**	**<0.001**	**-0.096 (-0.113; -0.079)**	**-0.166**	**<0.001**
Ln plank	**-0.323 (-0.368; -0.278)**	**-0.150**	**<0.001**	**-0.351 (-0.419; -0.283)**	**-0.156**	**<0.001**	**-0.293 (-0.352; -0.234)**	**-0.149**	**<0.001**
Sit and reach (cm)	**-0.011 (-0.014; -0.008)**	**-0.078**	**<0.001**	**-0.017 (-0.022; -0.012)**	**-0.100**	**<0.001**	**-0.007 (-0.011; -0.003)**	**-0.053**	**0.001**
Grip strength (kg)	**-0.008 (-0.011; -0.005)**	**-0.062**	**<0.001**	**-0.015 (-020; -0.011)**	**-0.114**	**<0.001**	0.00004 (-0.004; 0.004)	0.0004	0.984
CAMSA score	**-0.050 (-0.056; -0.043)**	**-0.159**	**<0.001**	**-0.065 (-0.076; -0.055)**	**-0.198**	**<0.001**	**-0.033 (-0.042; -0.024)**	**-0.116**	**<0.001**
PACER (laps completed)	**-0.015 (-0.017; -0.014)**	**-0.183**	**<0.001**	**-0.017 (-0.020; -0.015)**	**-0.219**	**<0.001**	**-0.012 (-0.014; -0.009)**	**-0.127**	**<0.001**
Fully adjusted models									
CAPL domain scores									
Knowledge and Understanding score	**-0.014 (-0.023; -0.005)**	**-0.032**	**0.003**	**-0.014 (-0.027; -0.0001)**	**-0.031**	**0.048**	**-0.015 (-0.028; -0.002)**	**-0.036**	**0.023**
Motivation and Confidence score	**-0.122 (-0.132; 0.112)**	**-0.274**	**<0.001**	**-0.146 (-0.160; -0.132)**	**-0.319**	**<0.001**	**-0.095 (-0.108; -0.082)**	**-0.228**	**<0.001**
Physical Competence score	**-0.022 (-0.028; -0.016)**	**-0.080**	**<0.001**	**-0.026 (-0.035; -0.018)**	**-0.095**	**<0.001**	**-0.015 (-0.024; -0.006)**	**-0.055**	**0.001**
Individual CAPL components									
Age (years)	**0.076 (0.053; 0.099)**	**0.075**	**<0.001**	**0.075 (0.039; 0.109)**	**0.069**	**<0.001**	**0.073 (0.043; 0.104)**	**0.081**	**<0.001**
Gender (boy=1, girl =2)	**-0.366 (-0.416; 0.316)**	**-0.366**	**<0.001**	-	-	-	-	-	-
CAPL site	-0.00001 (-0.00003; 0.00001)	-0.012	0.202	-0.00001 (-0.00003; 0.00002)	-0.008	0.553	-0.00002 (-0.00004; 0.00001)	-0.017	0.222
Waist circumference (cm)	0.002 (-0.001; 0.004)	0.016	0.206	0.002 (-0.002; 0.006)	0.013	0.452	0.002 (-0.002; 0.006)	0.019	0.288
Most time children should spend in front of a screen (hours/day)	**0.009 (0.009;0.010)**	**0.370**	**<0.001**	**0.009 (0.008; 0.010)**	**0.387**	**<0.001**	**0.009 (0.008; 0.010)**	**0.347**	**<0.001**
Frequency of PA (days/week)	**-0.071 (-0.083; -0.058)**	**-0.112**	**<0.001**	**-0.068 (-0.086; -0.050)**	**-0.105**	**<0001**	**-0.073 (-0.090; -0.057)**	**-0.127**	**<0.001**
Ln plank	**-0.090 (-0.139; -0.041)**	**-0.042**	**<0.001**	-0.023 (-0.097; 0.051)	-0.010	0.540	**-0.0159 (-0.224; -0.093)**	**-0.081**	**<0.001**
Sit and reach	-0.002 (-0.005; 0.001)	-0.014	0.177	**-0.006 (-0.011; -0.002)**	**-0.037**	**0.010**	0.001 (-0.003; 0.005)	0.009	0.539
2Grip strength (kg)	-0.001 (-0.004; 0.002)	-0.007	0.557	-0.003 (-0.008; 0.001)	-0.024	0.182	0.001 (-0.003; 0.005)	0.011	0.568
CAMSA score	**-0.012 (-0.019; -0.004)**	**-0.038**	**0.002**	**-0.016 (-0.027; -0.005)**	**-0.049**	**0.005**	-0.009 (-0.018; 0.001)	-0.030	0.078
PACER (laps completed)	**-0.006 (-0.008; -0.003)**	**-0.066**	**<0.001**	**-0.007 (-0.010; -0.040)**	**-0.088**	**<0.001**	-0.003 (-0.006; 0.001)	-0.028	0.106

Minimally adjusted models adjusted for age and gender. Standardized β for age and gender in minimally adjusted models ranged from 0.070 to 0.147 and -0.272 to -0.167, respectively. Fully adjusted models included all variables significant (*p<*0.10) in minimally adjusted models (as well as age and gender in the model of CAPL domains). Separate fully adjusted models were run for CAPL domains and individual CAPL components. Bolded variables were significant <0.10 in minimally adjusted models, and <0.05 in fully adjusted models. The fully adjusted models of CAPL domain scores and CAPL components accounted for 15% and 23%, respectively, of the total variance in computer and video game use in the full sample

*BMI* body mass index, *CAMSA* Canadian Agility and Movement Skill Assessment, *CAPL* Canadian Assessment of Physical Literacy, *CI* confidence interval, *Ln* natural log transformation, *PA* physical activity, *PACER* Progressive Aerobic Cardiovascular Endurance Run

**Table 7:** Correlates of total screen time (*n*=8307)

Variable	All participants			Boys (n=4143)			Girls (n=4164)		
	Unstandardized β (95% CI)	Standardized β	*p* value	Unstandardized β (95% CI)	Standardized β	*p* value	Unstandardized β (95% CI)	Standardized β	*p* value
Minimally adjusted models									
CAPL domain scores									
Total Physical Literacy	**-0.048 (-0.052; -0.045)**	**-0.277**	**<0.001**	**-0.057 (-0.062; -0.052)**	**-0.320**	**<0.001**	**-0.038 (-0.043; -0.033)**	**-0.223**	**<0.001**
Knowledge and Understanding score	**-0.078 (-0.093; -0.062)**	**-0.110**	**<0.001**	**-0.090 (-0.113; -0.067)**	**-0.125**	**<0.001**	**-0.063 (-0.084; -0.042)**	**-0.095**	**<0.001**
Motivation and Confidence score	**-0.214 (-0.229; -0.200)**	**-0.300**	**<.0001**	**-0.250 (-0.271; -0.229)**	**-0.340**	**<0.001**	**-0.172 (-0.192; -0.152)**	**-0.254**	**<0.001**
Physical Competence score	**-0.092 (-0.101; -0.082)**	**-0.204**	**<0.001**	**-0.107 (-0.121; -0.094)**	**-0.241**	**<0.001**	**-0.070 (-0.084; -0.057)**	**-0.158**	**<0.001**
Individual CAPL components									
Age (years)	**0.136 (0.101; 0.170)**	**0.083**	**<0.001**	**0.101 (0.048; 0.154)**	**0.058**	**<0.001**	**0.170 (0.126; 0.215)**	**0.115**	**<0.001**
Gender (boy=1, girl=2)	**-0.569 (-0.651; -0.488)**	**-0.148**	**<0.001**	**-**	**-**	**-**	**-**	**-**	**-**
CAPL site	**-0.0001 (-0.0001; -0.00002)**	**-0.033**	**0.002**	**-0.0001 (-0.0001; -0.00002)**	**-0.040**	**0.010**	**-0.00004 (-0.0001; 0.00001)**	**-0.026**	**0.095**
Season	-0.001 (-0.039; 0.037)	-0.0005	0.966	-0.017 (-0.075; 0.041)	-0.009	0.564	0.014 (-0.035; 0.063)	0.009	0.580
BMI *z*-score	**0.091 (0.059; 0.123)**	**0.060**	**<0.001**	**0.099 (0.053; 0.146)**	**0.065**	**<0.001**	**0.081 (0.038; 0.125)**	**0.056**	**<0.001**
Waist circumference (cm)	**0.016 (0.012; 0.020)**	**0.089**	**<0.001**	**0.017 (0.011; 0.023)**	**0.092**	**<0.001**	**0.015 (0.010; 0.020)**	**0.089**	**<0.001**
Most time children should spend in front of a screen (hours/day)	**0.017 (0.016; 0.017)**	**0.421**	**<0.001**	**0.017 (0.016; .018)**	**0.439**	**<0.001**	**0.017 (0.015; 0.018)**	**0.397**	**<0.001**
Frequency of PA (days/week)	**-0.167 (-0.188; -0.146)**	**-0.165**	**<0.001**	**-0.176 (-0.207; -0.144)**	**-0.168**	**<0.001**	**-0.157 (-0.185; -0.128)**	**-0.165**	**<0.001**
Ln plank	**-0.578 (-0.651; -0.505)**	**-0.167**	**<0.001**	**-0.632 (-0.741; -0.523)**	**-0.175**	**<0.001**	**-0.518 (-0.614; -0.422)**	**-0.161**	**<0.001**
Sit and reach (cm)	**-0.018 (-0.023; -0.013)**	**-0.079**	**<0.001**	**-0.028 (-0.0037; -0.020)**	**-0.104**	**<0.001**	**-0.011 (-0.017; -0.004)**	**-0.050**	**0.001**
Grip strength (kg)	**-0.013 (-0.018; -0.008)**	**-0.063**	**<0.001**	**-0.022 (-0.030; -0.015)**	**-0.104**	**<0.001**	-0.002 (-0.009; 0.004)	**-0.012**	0.479
CAMSA score	**-0.081 (-0.092; -0.070)**	**-0.161**	**<0.001**	**-0.107 (-0.124; -0.090)**	**-0.201**	**<0.001**	**-0.054 (-0.069; -0.039)**	**-0.116**	**<0.001**
PACER (laps completed)	**-0.026 (-0.029; -0.023)**	**-0.194**	**<0.001**	**-0.029 (-0.033; -0.025)**	**-0.227**	**<0.001**	**-0.021 (-0.025; -0.016)**	**-0.138**	**<0.001**
Fully adjusted models									
CAPL domain scores									
Knowledge and Understanding score	**-0.028 (-0.043; -0.013)**	**-0.039**	**<0.001**	**-0.032 (-0.054; -0.010)**	**-0.044**	**0.005**	**-0.024 (-0.045; -0.003)**	**-0.036**	**0.025**
Motivation and Confidence score	**-0.183 (-0.198; -0.167)**	**-0.255**	**<0.001**	**-0.210 (-0.233; -0.187)**	**-0.285**	**<0.001**	**-0.151; -0.173; -0.130**	**-0.223**	**<0.001**
Physical Competence score	**-0.045 (-0.055; -0.035)**	**-0.100**	**<0.001**	**-0.054 (-0.068; -0.40)**	**-0.121**	**<0.001**	**-0.031 (-0.045; -0.017)**	**-0.071**	**<0.001**
Individual CAPL components									
Age (years)	**0.099 (0.062; 0.136)**	**0.060**	**<0.001**	**0.074 (0.018; 0.130)**	**0.043**	**0.009**	**0.116 (0.068; 0.164)**	**0.078**	**<0.001**
Gender (boy=1, girl=2)	**-0.405 (-0.486; -0.325)**	**-0.105**	**<0.001**	-	-	-	-	-	-
CAPL site	-0.00003 (-0.0001; 0.000003)	-0.017	0.082	-0.00003 (-0.0001; 0.00002)	-0.016	0.255	-0.00003 (-0.0001; 0.00001)	-0.018	0.197
Waist circumference (cm)	0.004 (-0.0001; 0.009)	0.024	0.055	0.002 (-0.004; 0.009)	0.011	0.515	**0.007 (0.001; 0.013)**	**0.041**	**0.021**
Most time children should spend in front of a screen (hours/day)	**0.015 (0.015; 0.016)**	**0.393**	**<0.001**	**0.015 (0.014; 0.016)**	**0.400**	**<0.001**	**0.016 (0.015; 0.017)**	**0.380**	**<0.001**
Frequency of PA (days/week)	**-0.101 (-0.121; -0.082)**	**-0.100**	**<0.001**	**-0.084 (-0.117; -0.058)**	**-0.084**	**<0.001**	**-0.116 (-0.143; -0.090)**	**-0.123**	**<0.001**
Ln plank	**-0.196 (-0.0275; -0.117)**	**-0.057**	**<0.001**	-0.113 (-0.232; 0.005)	-0.031	0.059	**-0.283 (-0.388; -0.178)**	**-0.088**	**<0.001**
Sit and reach	-0.002 (-0.007; 0.003)	-0.010	0.363	**-0.010 (-0.018; -0.003)**	**-0.038**	**0.008**	0.004 (-0.002; 0.010)	0.018	0.214
Grip strength (kg)	-0.002 (-0.007; 0.003)	-0.010	0.440	-0.002 (-0.010; 0.005)	-0.010	0.571	-0.003 (-0.009; 0.004)	-0.014	0.443
CAMSA score	**-0.015 (-0.027; -0.003)**	**-0.030**	**0.014**	**-0.023 (-0.041; -0.005)**	**-0.044**	**0.011**	-0.009 (-0.024; 0.007)	-0.019	0.271
PACER (laps completed)	**-0.009 (-0.013; -0.006)**	**-0.070**	**<0.001**	**-0.012 (-0.017; -0.008)**	**-0.095**	**<0.001**	-0.005 (-0.010; 0.0004)	-0.031	0.072

Minimally adjusted models adjusted for age and gender. Standardized β for age and gender in minimally adjusted models ranged from 0.057 to 0.137 and -0.185 to -0.122, respectively. Fully adjusted models included all variables significant (*p<*0.10) in minimally adjusted models (as well as age and gender in the model of CAPL domains). Separate fully adjusted models were run for CAPL domains and individual CAPL components. Bolded variables were significant <0.10 in minimally adjusted models, and <0.05 in fully adjusted models. The fully adjusted models of CAPL domain scores and CAPL components accounted for 13% and 23%, respectively, of the total variance in total screen time in the full sample

*BMI* body mass index, *CAMSA* Canadian Agility and Movement Skill Assessment, *CAPL* Canadian Assessment of Physical Literacy, *CI* confidence interval, *Ln* natural log transformation, *PA* physical activity, *PACER* Progressive Aerobic Cardiovascular Endurance Run

**Table 8:** Correlates of non-screen sedentary behaviour (*n*=8307)

Variable	All participants			Boys (n=4143)			Girls (n=4164)		
	Unstandardized β (95% CI)	Standardized β	*p* value	Unstandardized β (95% CI)	Standardized β	*p* value	Unstandardized β (95% CI)	Standardized β	*p* value
Minimally adjusted models									
CAPL domain scores									
Total Physical Literacy	**-0.004 (-0.007; -0.002)**	**-0.038**	**0.001**	**-0.007 (-0.010; -0.003)**	**-0.060**	**<0.001**	-0.002 (-0.005; 0.002)	-0.013	0.406
Knowledge and Understanding score	**0.035 (0.024; 0.045)**	**0.074**	**<0.001**	**0.023 (0.008; 0.038)**	**0.050**	**0.002**	**0.049 (0.034; 0.064)**	**0.101**	**<0.001**
Motivation and Confidence score	**-0.037 (-0.047; -0.027)**	**-0.078**	**<0.001**	**-0.038 (-0.052; -0.024)**	**-0.082**	**<0.001**	**-0.035 (-0.050; -0.021)**	**-0.072**	**<0.001**
Physical Competence score	**-0.015 (-0.021; -0.008)**	**-0.050**	**<0.001**	**-0.020 (-0.029; -0.011)**	**-0.071**	**<0.001**	**-0.008 (-0.018; 0.002)**	**-0.026**	**0.100**
Individual CAPL components									
Age (years)	**0.131 (0.108; 0.154)**	**0.120**	**<0.001**	0.096 (0.063; 0.129)	0.088	<0.001	**0.166 (0.133; 0.198)**	**0.153**	**<0.001**
Gender (boy=1, girl=2)	**0.103 (0.049; 0.158)**	**0.040**	**<0.001**	-	-	-	-	-	-
CAPL site	0.00001 (-0.00001; 0.00003)	0.007	0.512	0.00002 (-0.00002; 0.00005)	0.15	0.320	-0.000002 (-0.00003; 0.00003)	-0.002	0.906
Season	**-0.027 (-0.053; -0.002)**	**-0.023**	**0.036**	-0.026 (-0.062; 0.011)	-0.022	0.165	**-0.030 (-0.065; 0.006)**	**-0.025**	**0.099**
BMI *z*-score	0.011 (-0.010; 0.033)	0.011	0.297	**0.026 (-0.003; 0.055)**	**0.027**	**0.082**	-0.005 (-0.037; 0.027)	-0.005	0.759
Waist circumference (cm)	**0.003 (0.0002; 0.006)**	**0.025**	**0.030**	**0.004 (0.004; 0.008)**	**0.035**	**0.032**	0.002 (-0.002; 0.006)	0.015	0.332
Most time children should spend in front of a screen (hours/day)	**0.003 (0.002; 0.004)**	**0.114**	**<0.001**	**0.002 (0.002; 0.003)**	**0.105**	**<0.001**	**0.004 (0.003; 0.005)**	**0.126**	**<0.001**
Frequency of PA (days/week)	**0.024 (0.001; 0.010)**	**0.036**	**0.001**	**0.024 (0.004; 0.043)**	**0.036**	**0.020**	**0.026 (0.013; 0.005)**	**0.038**	**0.013**
Ln plank	-0.040 (-0.089; 0.010)	-0.017	0.117	-0.056 (-0.125; 0.013)	-0.025	0.114	-0.019 (-0.090; 0.051)	-0.008	0.594
Sit and reach (cm)	0.0001 (-0.003; 0.004)	0.001	0.952	-0.001 (-0.007; 0.004)	-0.007	0.636	0.001 (-0.004; 0.005)	0.004	0.794
Grip strength (kg)	-0.002 (-0.006; 0.001)	-0.018	0.140	**-0.004 (-0.009; 0.0003)**	**-0.032**	**0.070**	-0.001 (-0.005; 0.004)	-0.004	0.807
CAMSA score	**-0.020 (-0.028; -0.013)**	**-0.061**	**<0.001**	**-0.031 (-0.041; -0.020)**	**-0.092**	**<0.001**	**-0.010 (-0.021; 0.001)**	**-0.030**	**0.070**
PACER (laps completed)	**-0.004 (-0.006; -0.002)**	**-0.044**	**<0.001**	**-0.004 (-0.007; -0.002)**	**-0.054**	**0.001**	**-0.003 (-0.006; 0.0002)**	**-0.029**	**0.063**
Fully adjusted models									
CAPL domain scores									
Knowledge and Understanding score	**0.046 (0.035; 0.057)**	**0.098**	**<0.001**	**0.035 (0.020; 0.049)**	**0.076**	**<0.001**	**0.059 (0.044; 0.075)**	**0.122**	**<0.001**
Motivation and Confidence score	**-0.040 (-0.051; -0.029)**	**-0.083**	**<0.001**	**-0.035 (-0.050; -0.020)**	**-0.075**	**<0.001**	**-0.043 (-0.059; -0.028)**	**-0.088**	**<0.001**
Physical Competence score	**-0.011 (-0.018; 0.004)**	**-0.036**	**0.003**	**-0.015 (-0.024; -0.006)**	**-0.054**	**0.002**	-0.006 (-0.016; 0.005)	-0.017	0.299
Individual CAPL components									
Age (years)	**0.130 (0.103; 0.157)**	**0.119**	**<0.001**	**0.104 (0.066; 0.142)**	**0.095**	**<0.001**	**0.153 (0.116; 0.190)**	**0.142**	**<0.001**
Gender (boy=1, girl=2)	**0.133 (0.077; 0.189)**	**0.052**	**<0.001**	-	-	-	-	-	-
Season	**-0.027 (-0.053; -0.002)**	**-0.023**	**0.034**	-0.022 (-0.059; 0.014)	-0.019	0.226	**-0.035 (-0.071; -0.00001)**	**-0.030**	**0.049**
Waist circumference (cm)	0.001 (-0.001; 0.004)	0.011	0.346	0.002 (-0.002; 0.006)	0.015	0.400	0.001 (-0.003; 0.005)	0.010	0.544
Most time children should spend in front of a screen (hours/day)	**0.003 (0.002; 0.004)**	**0.112**	**<0.001**	**0.002 (0.002; 0.003)**	**0.097**	**<0.001**	**0.004 (0.003; 0.005)**	**0.128**	**<0.001**
Frequency of PA (days/week)	**0.039 (0.025; 0.054)**	**0.058**	**<0.001**	**0.042 (0.022; 0.063)**	**0.064**	**<0.001**	**0.037 (0.016; 0.058)**	**0.053**	**0.001**
CAMSA score	**-0.015 (-0.023; -0.006)**	**-0.045**	**0.001**	**-0.026 (-0.038; -0.013)**	**-0.076**	**<0.001**	-0.006 (-0.018; 0.006)	-0.017	0.331
PACER (laps completed)	-0.002 (-0.004; 0.001)	-0.020	0.125	-0.001 (-0.004; 0.002)	-0.015	0.439	-0.002 (-0.006; 0.002)	-0.019	0.294

Minimally adjusted models adjusted for age and gender. Standardized β for age and gender in minimally adjusted models ranged from 0.098 to 0.141 and 0.029 to 0.047, respectively. Fully adjusted models included all variables significant (*p<*0.10) in minimally adjusted models (as well as age and gender in the model of CAPL domains). Separate fully adjusted models were run for CAPL domains and individual CAPL components. Bolded variables were significant <0.10 in minimally adjusted models, and <0.05 in fully adjusted models. The fully adjusted models of CAPL domain scores and CAPL components both accounted for 3% of the total variance in non-screen sedentary behaviour in the full sample

*BMI* body mass index, *CAMSA* Canadian Agility and Movement Skill Assessment, *CAPL* Canadian Assessment of Physical Literacy, *CI* confidence interval, *Ln* natural log transformation, *PA* physical activity, *PACER* Progressive Aerobic Cardiovascular Endurance Run

**Table 9:** Correlates of total sedentary behaviour (*n*=8307)

Variable	All participants			Boys (n=4143)			Girls (n=4164)		
	Unstandardized β (95% CI)	Standardized β	*p* value	Unstandardized β (95% CI)	Standardized β	*p* value	Unstandardized β (95% CI)	Standardized β	*p* value
Minimally adjusted models									
CAPL domain scores									
Total Physical Literacy	**-0.053 (-0.058; -0.048)**	**-0.232**	**<0.001**	**-0.064 (-0.071; -0.057)**	**-0.281**	**<0.001**	**-0.039 (-0.046; -0.032)**	**-0.170**	**<0.001**
Knowledge and Understanding score	**-0.043 (-0.063; -0.022)**	**-0.047**	**<0.001**	**-0.067 (-0.097; -0.038)**	**-0.073**	**<0.001**	-0.014 (-0.042; 0.015)	-0.015	0.339
Motivation and Confidence score	**-0.251 (-0.271; -0.232)**	**-0.270**	**<0.001**	**-0.288 (-0.315; -0.261)**	**-0.307**	**<0.001**	**-0.208 (-0.235; -0.181)**	**-0.225**	**<0.001**
Physical Competence score	**-0.106 (-0.119; 0.145)**	**-0.182**	**<0.001**	**-0.127 (-0.144; -0.110)**	**-0.225**	**<0.001**	**-0.078 (-0.097; -0.060)**	**-0.130**	**<0.001**
Individual CAPL components									
Age (years)	**0.267 (0.222; 0.312)**	**0.125**	**<0.001**	**0.197 (0.130; 0.264)**	**0.089**	**<0.001**	**0.336 (0.276; 0.397)**	**0.166**	**<0.001**
Gender (boy=1, girl=2)	**-0.466 (-0.573; -0.360)**	**-0.093**	**<0.001**	-	-	-	-	-	-
CAPL site	**-0.00004 (-0.0001; -0.000001)**	**-0.022**	**0.047**	-0.0001 (-0.0001; 0.00001)	-0.024	0.125	-0.00004 (-0.0001; 0.00002)	-0.020	0.195
Season	-0.028 (-0.078; 0.022)	-0.012	0.267	-0.043 (-0.117; 0.031)	-0.018	0.254	-0.016 (-0.082; 0.050)	-0.007	0.635
BMI *z*-score	**0.102 (0.060; 0.144)**	**0.052**	**<0.001**	**0.125 (0.066; 0.185)**	**0.064**	**<0.001**	**0.076 (0.018; 0.135)**	**0.039**	**0.011**
Waist circumference (cm)	**0.019 (0.014; 0.024)**	**0.081**	**<0.001**	**0.021 (0.014; 0.029)**	**0.090**	**<0.001**	**0.017 (0.010; 0.024)**	**0.073**	**<0.001**
Most time children should spend in front of a screen (hours/day)	**0.020 (0.019; 0.021)**	**0.382**	**<0.001**	**0.019 (0.018; 0.020)**	**0.397**	**<0.001**	**0.021 (0.019; 0.022)**	**0.359**	**<0.001**
Frequency of PA (days/week)	**-0.143 (-0.170; -0.115)**	**-0.109**	**<0.001**	**-0.152 (-0.192; -0.112)**	**-0.115**	**<0.001**	**-0.130 (-0.169; -0.092)**	**-0.101**	**<0.001**
Ln plank	**-0.617 (-0.713; -0.522)**	**-0.137**	**<0.001**	**-0.688 (-0.826; -0.549)**	**-0.149**	**<0.001**	**-0.537 (-0.668; -0.406)**	**-0.122**	**<0.001**
Sit and reach (cm)	**-0.018 (-0.025; -0.011)**	**-0.060**	**<0.001**	**-0.030 (-0.040; -0.019)**	**-0.086**	**<0.001**	**-0.010 (-0.019; -0.001)**	**-0.035**	**0.023**
Grip strength (kg)	**-0.015 (-0.022; -0.009)**	**-0.058**	**<0.001**	**-0.027 (-0.036; 0.017)**	**-0.097**	**<0.001**	-0.003 (-0.012; 0.006)	-0.011	0.513
CAMSA score	**-0.101 (-0.116; -0.087)**	**-0.155**	**<0.001**	**-0.137 (-0.0159; -0.116)**	**-0.204**	**<0.001**	**-0.064 (-0.084; -0.044)**	**-0.101**	**<0.001**
PACER (laps completed)	**-0.030 (-0.034; -0.026)**	**-0.171**	**<0.001**	**-0.033 (-0.038; -0.029)**	**-0.206**	**<0.001**	**-0.024 (-0.030; -0.018)**	**-0.117**	**<0.001**
Fully adjusted models									
CAPL domain scores									
Knowledge and Understanding score	0.018 (-0.002; 0.038)	0.020	0.075	0.003 (-0.026; 0.031)	0.003	0.858	**0.035 (0.007; 0.064)**	**0.039**	**0.015**
Motivation and Confidence score	**-0.222 (-0.243; -0.202)**	**-0.239**	**<0.001**	**-0.245 (-0.274; -0.215)**	**-0.261**	**<0.001**	**-0.195 (-0.224; -0.166)**	**-0.211**	**<0.001**
Physical Competence score	**-0.055 (-0.069; -0.042)**	**-0.095**	**<0.001**	**-0.069 (-0.087; -0.051)**	**-0.121**	**<0.001**	**-0.037 (-0.056; -0.017)**	**-0.061**	**<0.001**
Individual CAPL components									
Age (years)	**0.236 (0.186; 0.286)**	**0.111**	**<0.001**	**0.187 (0.114; 0.260)**	**0.085**	**<0.001**	**0.276 (0.209; 0.343)**	**0.137**	**<0.001**
Gender (boy=1, girl=2)	**-0.290 (0.397; -0.182)**	**-0.058**	**<0.001**	-	-	-	-	-	-
CAPL site	-0.00001 (-0.0001; 0.00003)	-0.006	0.521	0.000001 (-0.0001; 0.0001)	-0.0004	0.975	-0.00002 (-0.0001; 0.00003)	-0.013	<0.365
Waist circumference (cm)	**0.007 (0.01; 0.013)**	**0.030**	**0.019**	0.006 (-0.003; 0.014)	0.023	0.196	**0.009 (0.001; 0.017)**	**0.041**	**0.024**
Most time children should spend in front of a screen (hours/day)	**0.018 (0.017; 0.019)**	**0.359**	**<0.001**	**0.017 (0.016; 0.016)**	**0.363**	**<0.001**	**0.020 (0.018; 0.021)**	**0.0347**	**<0.001**
Frequency of PA (days/week)	**-0.062 (-0.089; -0.036)**	**-0.047**	**<0.001**	**-0.044 (-0.082; 0.006)**	**-0.033**	**0.022**	**-0.081 (-0.117; -0.044)**	**-0.062**	**<0.001**
Ln plank	**-0.154 (-0.260; -0.049)**	**-0.034**	**0.004**	-0.055 (-0.209; 0.098)	-0.012	0.480	**-0.246 (-0.392; -0.100)**	**-0.056**	**0.001**
Sit and reach	-0.0001 (-0.007; 0.006)	-0.0004	0.973	-0.009 (-0.019; 0.001)	-0.025	0.088	0.005 (-0.003; 0.014)	0.019	0.204
Grip strength (kg)	-0.004 (-0.011; 0.003)	-0.015	0.258	-0.005 (-0.015; 0.005)	-0.019	0.304	-0.004 (-0.014; 0.005)	-0.016	0.401
CAMSA score	**-0.031 (-0.047; -0.015)**	**-0.048**	**<0.001**	**-0.050 (-0.074; -0.027)**	**-0.074**	**<0.001**	-0.016 (-0.038; 0.005)	-0.026	0.138
PACER (laps completed)	**-0.012 (-0.016; -0.017)**	**-0.066**	**<0.001**	**-0.014 (-0.020; -0.008)**	**-0.085**	**<0.001**	**-0.007 (-0.014; -0.000004)**	**-0.034**	**0.049**

Minimally adjusted models adjusted for age and gender. Standardized β for age and gender in minimally adjusted models ranged from 0.102 to 0.178 and -0.124 to -0.075, respectively. Fully adjusted models included all variables significant (*p<*0.10) in minimally adjusted models (as well as age and gender in the model of CAPL domains). Separate fully adjusted models were run for CAPL domains and individual CAPL components. Bolded variables were significant <0.10 in minimally adjusted models, and <0.05 in fully adjusted models. The fully adjusted models of CAPL domain scores and CAPL components accounted for 10% and 19%, respectively, of the total variance in total sedentary behaviour

*BMI* body mass index, *CAMSA* Canadian Agility and Movement Skill Assessment, *CAPL* Canadian Assessment of Physical Literacy, *CI* confidence interval, *Ln* natural log transformation, *PA* physical activity, *PACER* Progressive Aerobic Cardiovascular Endurance Run

In contrast to screen-based modes of SB, a smaller number of correlates displayed significant associations with non-screen SB. CAPL site, plank, and sit and reach were not significantly associated with non-screen SB in the group as a whole, or in either gender when examined separately. Non-screen SB was negatively associated with grip strength, and positively associated with BMI *z*-score in boys only. Season of data collection was associated with non-screen SB in girls and the group as a whole, but not in boys when examined separately.

### Correlates in fully adjusted model

#### CAPL domain scores

In the fully adjusted model, Physical Competence and Motivation and Confidence were negatively associated with all modes of SB in the sample as a whole, which was also generally true when examining boys and girls separately (Tables 5-9). Motivation and Confidence was the strongest correlate of all screen-based modes of SB (standardized β’s: -0.274 to -0.083, all *p<*0.05). Knowledge and Understanding was negatively associated with all screen-based modes of SB (standardized β’s: -0.039 to -0.032, all *p<*0.05); however, it was positively associated with non-screen SB (standardized β: 0.098, *p<*0.05), and there was no significant association observed for total SB (standardized β: 0.020, *p>*0.05).

#### Individual CAPL components

Correlates were similar among all modes of screen-based SB, although there were important differences when comparing screen and non-screen SB. The self-reported maximum amount of time that participants felt children should spend in front of a screen each day was positively associated with all modes of SB in the full sample (standardized β’s: 0.112 to 0.393, all *p<*0.05). Log-transformed plank score and PACER score were negatively associated with all screen-based modes of SB, while CAMSA score was negatively associated with all forms of SB other than TV viewing (all *p<*0.05). Associations were generally similar when examining boys and girls separately, although in girls the CAMSA score was not independently associated with any mode of SB, and PACER was independently associated only with total SB. Gender and self-reported PA were positively associated with non-screen SB (indicating higher levels for girls), and negatively associated with all modes of screen-based SB (all *p<*0.05). Age was positively associated with all modes of SB other than TV viewing (*p*=0.051). Grip strength and sit and reach were not associated with any mode of SB in the fully adjusted model (all *p>*0.05).

## Discussion

The purpose of the present study was to identify whether aspects of PL were associated with key modes of SB among children participating in the RBC-CAPL Learn to Play study. Our results demonstrate significant correlations between common modes of SB and important aspects of PL. Further, our results suggest that the relationship between PL and SB differ based on the mode of SB being examined. We observed that Physical Competence and Motivation and Confidence were negatively associated with all modes of SB in the group as a whole, with the largest β coefficients observed for Motivation and Confidence. In the fully adjusted models, a 1-point increase in Motivation and Confidence was associated with 13 minutes/day less total SB, while a similar increase in Physical Competence was associated with 3 minutes/day less total SB. These results suggest that although all CAPL domains are related to important modes of SB, targeting Motivation and Confidence may offer the best means of intervening on SB (or vice versa).

Similar findings were observed for individual CAPL components. Plank and PACER scores were negatively associated with screen-based SB and total SB, but not non-screen SB. Self-reported PA was positively associated with non-screen SB, and negatively associated with all other modes of SB. The fully adjusted model of individual CAPL components accounted for 23% of the variance in screen-based SB, but just 3% of the variance in non-screen SB. Among screen-based SB, CAPL components also accounted for a greater proportion of the variance in computer and video game use (23%), when compared to TV viewing (11%). These results suggest that PL is more strongly negatively associated with screen-based modes of SB, and especially computer and video game use, rather than non-screen SB.

These results are supported by other recent findings that have also shown contrasting correlates for screen-based and non-screen SBs. For example, a recent systematic review by Carson et al. [4] concluded that reading time was not consistently associated with any physical health indicator, whereas screen-based SBs were associated with unfavourable measures of body composition, aerobic and musculoskeletal fitness, cardiometabolic health, prosocial behaviour, and self-esteem. In contrast to screen-based SBs, our results demonstrate that there may be differential effects associated with non-screen SB.

Knowledge and Understanding and self-reported PA were both negatively associated with screen-based modes of SB, but positively associated with non-screen SB. The examples provided for non-screen SB in the self-report questionnaire were reading, homework, talking to friends, and drawing. It is perhaps not surprising that non-screen SB was positively associated with the Knowledge and Understanding domain of PL, given that reading and homework could expose children to important concepts related to PL, physical activity, and health. The magnitude of this association, however, was small; a 1-unit higher Knowledge and Understanding score was associated with a 3 minute/day increase in non-screen SB.

The positive association between non-screen-based SB and the frequency of PA is more difficult to explain, although not unprecedented. For example, in 10,900 American adults, Dunton et al. [29] found that PA was positively associated with time spent reading. It is possible that this relationship is mediated through increased PL-related Knowledge and Understanding. The current study did not assess parental socio-economic status, which is positively associated with reading achievement [30, 31]. It is therefore plausible that non-screen SBs could serve as a proxy for socio-economic status, which is also associated with childhood PA [32]. Previous work has shown that certain health-related behaviours tend to cluster together [33], which could contribute to the associations observed in the present study as well. As with Knowledge and Understanding, the clinical significance of this relationship is questionable; an extra day/week of PA was associated with less than 1 minute/day higher non-screen SB. Nonetheless, these findings suggest that time spent in non-screen SB may not adversely affect time spent in PA, and is likely preferable to time spent in other forms of SB. Future research should further investigate the association between non-screen-based SB and PA in this age group, as well as comparing the health impacts of different combinations of screen and non-screen SB.

The most consistent positive correlate of all modes of SB was a question asking children to select the maximum amount of time a child should spend using screens each day. Children who indicated a higher acceptable level for daily screen time had higher levels for each mode of SB, in both the group as a whole and in boys and girls separately. Previous research indicates that children who have rules related to screen time tend to watch less TV [34, 35]. Not surprisingly, children whose parents accumulate large amounts of screen time are more likely to get more screen time themselves [34, 35]. It is plausible that children who believe 2-4 hours/day is an acceptable level of screen time, live in households with fewer rules and/or less positive role modelling related to SB. However, the magnitude of the association for this correlate was small. Based on the fully adjusted model, children who selected the lowest acceptable amount of screen time (30 minutes/day) would be expected to have just 3 minutes/day less screen time than those who indicated the highest value (4 hours/day). Although this association is novel, our results indicate that simply educating children on acceptable levels of screen time may not result in a large reduction in daily SB.

Similar to previous research, our findings suggest that SB increases with age [36]. In the fully adjusted models, we saw that each 1-year increase in age was associated with a 14-minute increase in total SB, with smaller increases observed for individual modes of SB. Future interventions should therefore explore ways to minimize age-related increases in SB.

When examining both genders combined, waist circumference was associated with all modes of SB in the minimally adjusted model, but only with total SB in the fully adjusted model. Among girls (but not boys), waist circumference was associated with TV viewing and total screen time. Waist circumference was not associated with computer/video game use or non-screen SB in either gender. This is in contrast with previous research, which has generally found consistent associations between screen time (especially TV viewing) and markers of body composition in both genders [4, 11]. There has, however, been evidence for gender-based differences in the associations between waist circumference and SB modalities in this age group. A previous study by our group [37] found that waist circumference was independently associated with TV viewing in girls only, and with computer time in boys only. It is unclear why waist circumference was not associated with any mode of SB in boys in the present study. It may be because previous research did not adjust for direct measures of cardiorespiratory and musculoskeletal fitness, which were more consistently associated with all modes of SB in the present analysis. This is supported by a recent factor analysis that concluded that body composition does not contribute significantly to the total CAPL score [38].

In the present analysis, season of data collection was independently associated with non-screen SB, indicating less non-screen SB as the year progressed from winter through fall. However, the magnitude of this relationship was small – a change in season was associated with just a 1.6 minute/day reduction in non-screen SB. Further, there was no significant relationship observed between season of data collection and any screen-based mode of SB. These findings are supported by previous research, which has generally found little or no relationship between season and SB in other samples of children and youth [39–41]. This is in contrast to PA, which has been shown to decrease during periods of cold and/or wet weather [42, 43]. These findings suggest that self-reported SB, especially screen-based SB, is relatively consistent throughout the year among this age group.

In the present study, 54% of children reported meeting the Canadian screen time guidelines of ≤2 hours/day of recreational screen time. This is similar to the prevalence of 10 year-old Canadian children who met these guidelines in the International Study of Childhood Obesity, Lifestyle and the Environment (ISCOLE) [11]. We observed no clear patterns with respect to regional differences in total SB or meeting the screen guidelines, and individual CAPL sites were not associated with any specific mode of SB in the fully adjusted model. In line with previous research [11, 37], boys accumulated higher levels of TV, computer time, total screen time, and total SB, while girls accumulated more non-screen-SB; as well, girls were more likely (61% vs 48%) to report meeting Canada’s screen time guidelines. These findings illustrate a generally healthier pattern of SB among girls compared to boys. These results highlight important gender differences in the pattern of SB, and suggest that SB interventions and public health strategies should be tailored depending on the age, gender, interests, and baseline habits of participants.

The present study employed linear regression models to investigate correlates of SB. Now that potential correlates have been identified, other techniques (e.g., structural equation modelling) may be useful to further investigate relationships between correlates and SB in future studies. This may be especially useful with respect to the Knowledge and Understanding and Motivation and Confidence domains, which rely on self-reported information, and would lend themselves to this approach.

### Strengths and limitations

The present study obtained a large sample size (>8,000 participants), collected from 11 sites across Canada, with roughly equal numbers of boys and girls. However, the number of participants approached for participation was not recorded. Although there were some statistically significant differences between those with missing data when compared to those with complete data, the magnitude of the differences was small and unlikely to be of clinical significance. To date this study is the largest examination of PL in this age group, used validated measurement tools, and included directly measured fitness, body composition, and motor performance. However, all modes of SB were self-reported, as was the frequency of PA participation, and this type of reporting can increase error and bias [44] when compared to objective measures. The current study investigated both screen and non-screen modes of SB, although screen use focused exclusively on TV, computer, and video game use. We therefore were unable to investigate the relationship between PL and other types of screen use, including tablets and smartphones. In addition, a cross-sectional design was employed, and therefore this study cannot be used to infer causality. Further, although we noted several independent correlates of SB, the magnitude of individual associations was small in the fully adjusted models. Finally, the present study did not assess socio-demographic variables such as income, parental education, or family structure, and therefore could not evaluate whether these variables would influence the relationship between PL and SB.

## Conclusions

Our findings show that key modes of SB are associated with total PL, as well as the Motivation and Confidence, Knowledge and Understanding, and Physical Competence domains. Motivation and Confidence demonstrated the strongest association with screen-based modes of SB, while Knowledge and Understanding showed positive associations with non-screen SB, and negative associations with screen-based SB.

In the fully adjusted model, the self-reported amount of time that participants felt that children should spend in front of a screen each day was positively associated with all modes of SB, while PACER and log-transformed plank scores were negatively associated with screen-based SB. Self-reported PA was negatively associated with screen-based modes of SB, and positively associated with non-screen SB. These results highlight the important differences between screen and non-screen SB, and suggest that public health interventions should continue to target screen-based SBs, given their deleterious associations with important aspects of PL. Interventions attempting to reduce screen-based SB may benefit from increasing children’s Motivation and Confidence, given the consistent and independent associations observed in the present analysis. Promotion of non-screen SB may have small benefits for some aspects of PL (Knowledge and Understanding, and self-reported PA), but negative changes for others (total PL, Motivation and Competence, and Physical Competence). Finally, interventions should be tailored to participant gender and age, which are associated with multiple modes of SB in this age group.

## Abbreviations

BMI: body mass index
CAMSA: Canadian Agility and Movement Skill Assessment
CAPL: Canadian Assessment of Physical Literacy
CSAPPA: Children’s Self-Perception of Adequacy in and Predilection for Physical Activity
Ln: natural log transformation
MET: metabolic equivalent
PA: physical activity
PACER: Progressive Aerobic Cardiovascular Endurance Run
PL: physical literacy
RBC-CAPL: Royal Bank of Canada–Canadian Assessment of Physical Literacy
SB: sedentary behaviour
